# Health risk assessment of metals in chicken meat and liver in Egypt

**DOI:** 10.1007/s10661-023-11365-9

**Published:** 2023-06-02

**Authors:** Heba F. Kamaly, Ahmed A. Sharkawy

**Affiliations:** Department of Forensic Medicine and Toxicology, Faculty of Veterinary Medicine, Assiut University, Assiut, Egypt

**Keywords:** Heavy metals, Poultry edibles, Estimated daily intake, Target hazard quotient, Hazard index, Target cancer risk

## Abstract

This study aimed to evaluate the concentration of metals such as aluminum (Al), cadmium (Cd), lead (Pb), barium (Ba), bismuth (Bi), cobalt (Co), nickel (Ni), chromium (Cr), iron (Fe), copper (Cu), zinc (Zn), and selenium (Se) in 360 samples of poultry meat and liver from six brands (A, B, C, D, E, and F) in Assiut, Egypt; compare these concentrations with Egyptian and world permissible limits; and determine their safety for human consumption according to health risk assessment. Chest, thigh muscles, and liver were collected randomly from Assiut city markets, and the concentration of heavy metals was measured in the central laboratory of the faculty of agriculture at Assiut University using inductively coupled plasma mass spectrometry (ICP-MS). All the analyzed samples were positive for the tested metals and were far below the allowed maximum permissible limits except for Pb and Fe, which exceeded the Egyptian Organization for Standardization (EOS) permissible limits with 33% and 67%, respectively, as well as Pb and Cd, which exceeded FAO/WHO permissible limits with 94% and 17%, respectively. Health risk assessment revealed the safety and minimum health risk for human consumption of metal residues in poultry tissues and liver using estimated daily intake (EDI), target hazard quotient (THQ), hazard index (HI), and target cancer risk (TR). Even though the THQ and HI values were significantly lower than 1.0 during our study, heavy metal monitoring in poultry products and byproducts is required for human security and safety.

## Introduction

Heavy metal pollution in food and feed is increasing every day due to anthropogenic activities in industry and agriculture. Poultry are exposed to a massive array of metals through contaminated feed, drinking water, and litter, which can reduce the safety of their food products (Ahmed, 2003). Pollution occurs through different steps in industries that enter the food chain and contaminate our food (Gholizadeh et al., 2009; Khairy, 2009).

Poultry meat has high nutritional value thanks to its essential amino acids, vitamins, minerals, important trace elements, and antioxidants, which promote human health and growth, especially in developing countries (Hassanin et al., 2014; Taha, 2003). All over the world, the poultry industry is well developed due to its easy digestibility, acceptance, and relative cheapness compared with other meat products. Contamination of poultry feed with heavy metals has an effect not only on livestock health, production, and reproduction but also on the safety of its products (Peng et al., 2018).

Heavy metal residues are characterized by no taste or smell, persistence, bioaccumulation in tissues, biomagnification in the food chain, different toxic effects, and being hidden in meat and offal (Darwish et al., 2018; Demirezen & Uruç, 2006; Ragab et al., 2014). The accumulation of heavy metals varies by organ in the same animal or bird depending on the consumed dose, exposure interval, animal breed, and age (John & Jeanne, 1994). Estimation of heavy metals in poultry edibles must receive further serious attention (Iwegbue et al., 2008; Ramadan & Adam, 2007) due to their bioaccumulation in living tissues and further toxic effects on vital body systems (Ahmed et al., 2019; Devi & Yadav, 2018). Heavy metals in food are considered a risk, so health institutions and organizations set standardization limits and monitoring programs to adjust permissible and non-permissible limits of heavy metals in food (Andrée et al., 2010; EC, 2003, 2008). Metals are classified as essential metals, such as Fe, Cu, Zn, and Se, which play a role in the structure of vitamins and enzymes but can be harmful if consumed in excess (Filazi et al., 2003; Niedziółka et al., 2010), and heavy metal pollutants, which can cause toxic effects even at low levels, such as Al, Cd, and Pb (Lenntech, 2012).

Al is a neurotoxic metal, and there is a relationship between it and Alzheimer's disease (Alfrey et al., 1976). Food is the main source of Al intake due to its wide use in our lives. It has been classified as a non-carcinogenic metal in "Group 3" by the International Agency for Research on Cancer (IARC) (EPA, 2004; IARC, 2012) and the Joint FAO/WHO Expert Committee on Food Additives (JECFA) and the Scientific Committee for Food (SCF) set 7.00 mg/kg body weight per week as a tolerable weekly intake (PTWI) for Al (JECFA, 1989).

Cd is an endocrine disruptor metal that causes renal and pulmonary dysfunction, bone defects, and myocardial diseases in animals (Akan et al., 2010; Faten et al., 2014; McLaughlin et al., 1999). The IARC classified Cd as a human carcinogen in "Group 1" (EFSA, 2009). According to the EU, FAO/WHO, and Turkey, the maximum permissible limits of Cd are 0.05 and 0.5 mg/kg in poultry meat and liver, respectively (FAO/WHO, 2006; EC, 2008; TFC, 2008), and the EOS set 0.5 mg/kg as the guideline limit of Cd (EOS, 1993).

Pb can enter the body through inhalation or ingestion and cause disturbances in different body systems (Berny et al., 1994). It has been classified as a "Group 2B" potential human cancer by the IARC (IARC, 2012). According to the EU and FAO/WHO, the maximum residue limits for Pb in poultry meat and liver are 0.1 and 0.5 mg/kg, respectively (FAO/WHO, 2006; EC, 2008). The EOS also suggested 2 mg/kg as a guideline for Pb in food (EOS, 1993).

Co enters the food chain in the form of fertilizers, is absorbed by plants, and then reaches the animal body, where it threatens lung, heart, and thyroid functions (Bucher et al., 1990; Coates JR & Watson, 1971; Pier, 1975). According to IARC, it has been classified as "probably carcinogenic" to humans in "Group 2A" (IARC, 1999, 2006).

Ni disrupts respiratory, cardiovascular, and nervous system functions in the body (Macomber & Hausinger, 2011). It is lethal if it exceeds the permissible limit in edibles (Nriagu & Pacyna, 1988). According to IARC, it has been classified as a human carcinogen in "Group 1" (IARC, 1990, 2012). The Food and Nutrition Board (FNB, 2010) set the permissible limit of Ni at 4 mg/kg, but the EOS showed that the safe guideline for Ni in food is 10 mg/kg wet weight (EOS, 1993).

Cr (III) acts as a cofactor of insulin in the body, so it has a vital role in glucose, lipid, and protein metabolism. Cr (IV) is considered a carcinogen (Wilbur et al., 2012), and the IARC confirms that and classifies it as a human carcinogen in "Group 1" (IARC, 1990, 2012). The World Health Organization and the Food and Agriculture Organization of the United Nations set 1 mg/kg as a limit for Cr exposure (WHO/FAO, 2011). Finally, Ba, Bi, Cu, Zn, Fe, and Se have been classified by the IARC as "Group 3" non-carcinogenic metals (EPA, 2004; IARC, 2012).

In last year's, renal failure, liver cirrhosis, chronic anemia, and other health problems have increased in the Egyptian population due to exposure to metals (Salem et al., 2000), and about 25% of human diseases today are due to long-term exposure to environmental pollution (UNEP, 2008).

The purpose of this study was to determine the level of safety of heavy metals (Al, Cd, Pb, Ba, Bi, Co, and Ni) and essential metals (Cr, Fe, Cu, Zn, and Se) residues in poultry chest, thigh muscles, and liver from six brands in Assiut, Egypt, using EDI, THQ, HI, and TR health risk assessments.

## Materials and Methods

### Sample preparation and analysis

Three hundred and sixty chest, thigh, and liver samples were randomly collected from Assiut city markets from six brands, 20 samples per brand. Samples were labeled, stored in a polyethylene bag, and frozen at -20 °C until the time of analysis. All laboratory containers were washed with a 10% solution of nitric acid prior to use. About one gram of each sample was individually soaked in 5 ml of high-grade nitric acid (68%; Merck, Germany) over night at room temperature as a digestive solution. The solution was heated at 150 °C for 5 h until the brown vapors disappeared and the sample solution became colorless. The residual solutions (~ 1.5–2 ml) were allowed to cool at room temperature, washed with 25 ml of distilled water, filtrated with ashless No. 42 filter paper, and stored in the freezer until analysis for heavy metals.

The concentration of each metal was determined by inductively coupled plasma mass spectrometry (ICP-MS, iCAP 6000 series) in the central laboratory of the faculty of agriculture at Assiut University against standard solutions of each metal, which were purchased from Sigma, USA.

For assurance quality control of analysis, procedural blanks and duplicate of the samples were done in each batch of digested samples. In addition, a recovery of the total analytical procedure was processed for metals in poultry edibles by spiking the analyzed samples with different standard concentrations. In order to ensure the reliability of instruments, a blank and known standard were run after every 10 samples. Accepted recoveries % were 97.3, 99.6, 93.6, 105.3, 103.16, 105.6, 92.1, 104.9, 104.5, 99.5, 97.6, and 100.46 for Al, Cd, Pb, Ba, Bi, Co, Ni, Cr, Fe, Cu, Zn, and Se, respectively. Metal concentrations were interpreted in µg/g (ppm).

## Statistical analysis

Using the SPSS program for Windows, version 16.0, for expressing heavy metal concentrations as the mean ± SE. The results were analyzed statistically using one-way analysis of variance (ANOVA) with Tukey and Dunnett multiple comparison tests.

## Health risk assessment

Health risks for consumption of metal residues in poultry tissues and liver were assessed based on calculations of EDI, THQ, HI, and TR.

The EDI was calculated according to the United States Environment Protection Agency (USEPA) (2010) as the following equation:

$$\mathrm{EDI}=\frac{(\mathrm{C}\times \mathrm{FIR})}{\mathrm{BW}}$$

where C is the concentration of the metal in each sample (µg/g). FIR is the rate of ingestion of poultry meat (muscles) in Egypt, which was estimated to be 39.53 g/day (FAOSTAT, 2013) and 0.1 g/day for liver tissue (WHO, 2003). BW is the body weight of the Egyptian adults, which was set at 70 kg.

According to the USEPA (1989), the non-cancer risk of heavy metals was calculated as follows:

$$\mathrm{THQ}=\frac{(\mathrm{ED}\times \mathrm{C}\times \mathrm{FIR}\times \mathrm{EF})}{(\mathrm{RfD}\times \mathrm{BW}\times \mathrm{AT})}\times {10}^{-3}$$

where THQ is the target hazard quotient; ED is exposure duration, which equals 30 years for non-cancer risk and 70 years for cancer risk as suggested by the USEPA; C represents the metal concentration in poultry meat (µg/g); FIR represents the food ingested rate (g/day); EF represents the exposure frequency (365 days/year); and RfD represents the metal reference dose (mg/kg/day), which represents the daily oral exposure dose of metal with the human population over a lifetime without an appreciable risk of deleterious effects (Akoto et al., 2014). RfD values for Al, Cd, Pb, Ba, Bi, Co, Ni, Cr, Fe, Cu, Zn, and Se are 0.0004, 0.001, 0.07, 0.00029, 0.0003, 0.02, 0.003, 0.7, 0.005, 0.3, and 0.005 (µg/g BW/day), respectively (USEPA, 2000, 2006). BW is the body weight of the Egyptian adults, and AT is the average exposure time (365 days × exposure years, which are set at 70 years). If the ratio is equal to or greater than 1, the Egyptian population will be at risk from these poultry edibles (Chien et al., 2002).

Hazard indexes aid in assessing the risk of combined metal exposure using the following equation:

$$\mathrm{HI}=\sum \mathrm{HQ}$$

In our study, HI = HQ _Al +_ HQ _Cd +_ HQ _Pb +_ HQ _Ba +_ HQ _Bi +_ HQ _Co +_ HQ _Ni +_ HQ _Cr +_ HQ _Fe +_ HQ _Cu +_ HQ _Zn +_ HQ _Se_ (USEPA, 1989). where ∑HQ means the sum of the hazard quotients of metals in the study. When HI exceeded 1, there was concern for a potential health effect (Huang et al., 2008).

Target cancer risk was used to indicate the carcinogenic risk of heavy metal residues in poultry edibles. It was estimated, according to USEPA (2018), as the following equation:

$$\mathrm{TR}=\frac{(\mathrm{ED}\times \mathrm{EF}\times \mathrm{FIR}\times \mathrm{C}\times \mathrm{CPSo})}{(\mathrm{ABW}\times \mathrm{AT})}\times {10}^{-3}$$

where TR is the target cancer risk; ED is exposure duration, which equals 70 years for the cancer risk suggested by the USEPA; EF is exposure frequency (365 days/year); FIR is the food ingested rate (g/day); C_M_ is the concentration of metal in poultry meat (µg/g); CPSo is the carcinogenic potency slope, oral (mg/kg body weight/day). The CPSo values are 0.38, 0.0085, 0.7, 1.7, and 0.5 for Cd, Pb, Co, Ni, and Cr, respectively (USEPA, 2018). The average body weight (ABW) is 70 kg for Egyptian adults, and AT_c_ is the average time for carcinogens is 365 days per year for 70 years.

## Results and Discussion

### Metal concentrations in poultry muscles and liver

The concentration mean, standard error, and range of heavy metals (Al, Cd, Pb, Ba, Bi, Co, and Ni) and essential metals (Cr, Fe, Cu, Zn, and Se) were analyzed in the muscles and liver of six brands of poultry sold in Assiut province markets. Results were discussed and compared with available national and international maximum permissible limits.

Among the analyzed heavy metals in poultry edibles, Al was the most highly concentrated metal; its range was 8.610–21.985 µg/g in chest muscles, 5.729–18.533 µg/g in thigh muscles, and 5.873–14.005 µg/g in the liver of six brands (Table 1). The obtained results for Al were in agreement with the study conducted by Mahmoud and Abdel-Mohsein (2015), where Al was the highest analyzed metal with concentrations of 8.44 and 16.44 µg/g in liver and 7.74 and 10.1 µg/g in muscles of Assiut and Qena broiler farms, respectively, and Wang et al. (2020), where the Al mean was 5.199 µg/g in broilers, but disagreed with the study done by Korish and Attia (2020), where undetectable Al levels were found in different poultry meat products and liver.

**Table 1:** Heavy metal residual concentrations mean (µg/g) in poultry chest, thigh muscles, and liver of six brands (*n* = 20 each)

			Brands					
Heavy metal	Organ	µg/g	1	2	3	4	5	6
Al	Chest Thigh Liver	Mean ± S.E	11.535 ± 0.160	8.610 ± 0.116	12.361 ± 0.221	21.985 ± 0.329	16.357 ± 0.356	10.945 ± 0.218
			12.808 ± 0.111a	5.729 ± 0.074a	18.533 ± 0.382a	12.101 ± 0.208a	16.357 ± 0.355	10.017 ± 0.234a
			5.873 ± 0.074ab	9.635 ± 0.126ab	13.014 ± 0.271ab	14.005 ± 0.276ab	10.935 ± 0.285ab	10.154 ± 0.162ab
Cd	Chest Thigh Liver	Mean ± S.E	0.054 ± 0.001	0.014 ± 0.001	0.029 ± 0.001	0.031 ± 0.001	0.024 ± 0.001	0.036 ± 0.001
			0.052 ± 0.001	0.088 ± 0.001a	0.019 ± 0.001a	0.025 ± 0.001a	0.015 ± 0.001a	0.024 ± 0.001a
			0.104 ± 0.002ab	0.037 ± 0.001ab	0.047 ± 0.002ab	0.041 ± 0.003ab	0.027 ± 0.0002ab	0.039 ± 0.003ab
Pb	Chest Thigh Liver	Mean ± S.E	2.577 ± 0.071	3.152 ± 0.154	2.560 ± 0.104	2.589 ± 0.090	3.301 ± 0.122	5.552 ± 0.322
			1.082 ± 0.006a	0.334 ± 0.001a	0.794 ± 0.007a	1.003 ± 0.011a	0.728 ± 0.005a	0.525 ± 0.002a
			0.146 ± 0.001ab	0.952 ± 0.001ab	0.936 ± 0.003ab	0.659 ± 0.004ab	0.733 ± 0.004ab	0.665 ± 0.006ab
Ba	Chest Thigh Liver	Mean ± S.E	6.226 ± 0.110	2.828 ± 1.443	2.839 ± 0.043	2.520 ± 0.059	3.211 ± 0.050	1.971 ± 0.050
			5.007 ± 0.106a	3.216 ± 0.026a	6.779 ± 0.090a	5.559 ± 0.163a	3.532 ± 0.107a	4.151 ± 0.116a
			1.038 ± 0.018ab	4.369 ± 0.112ab	3.122 ± 0.105ab	3.807 ± 0.070ab	1.529 ± 0.013ab	4.416 ± 0.107ab
Bi	Chest Thigh Liver	Mean ± S.E	3.526 ± 0.234	1.363 ± 0.090	0.873 ± 0.057	0.587 ± 0.038	0.719 ± 0.046	0.687 ± 0.045
			0.0063 ± 0.0001a	0.0043 ± 0.0004a	0.0069 ± 0.0001a	0.0133 ± 0.0003a	0.0053 ± 0.0001a	0.0084 ± 0.0001a
			0.011 ± 0.0001ab	0.0103 ± 0.001ab	0.0107 ± 0.0001ab	0.009 ± 0.0001ab	0.001 ± 0.0001ab	0.0102 ± 0.0001ab
Co	Chest Thigh Liver	Mean ± S.E	0.055 ± 0.001	0.052 ± 0.001	0.051 ± 0.001	0.083 ± 0.003	0.039 ± 0.001	0.067 ± 0.002
			0.039 ± 0.001a	0.014 ± 0.001a	0.039 ± 0.001a	0.037 ± 0.001a	0.033 ± 0.001a	0.021 ± 0.001a
			0.045 ± 0.002ab	0.053 ± 0.001ab	0.040 ± 0.003a	0.046 ± 0.0001a	0.028 ± 0.002ab	0.029 ± 0.001ab
Ni	Chest Thigh Liver	Mean ± S.E	0.228 ± 0.001	0.154 ± 0.001	0.177 ± 0.001	0.180 ± 0.001	0.182 ± 0.002	0.214 ± 0.002
			0.255 ± 0.004a	0.143 ± 0.002a	0.157 ± 0.001a	0.145 ± 0.001a	0.189 ± 0.001a	0.163 ± 0.001a
			0.217 ± 0.001ab	0.112 ± 0.001ab	0.173 ± 0.001ab	0.188 ± 0.003ab	0.174 ± 0.001ab	0.179 ± 0.001ab

The data is represented by the mean of heavy metal concentrations ± S.E. in the chest, thigh muscles, and liver, as well as their significant difference, where (a) indicates significant differences with the chest muscles and (b) indicates significant differences with the thigh muscles at *p* < 0.05

Human and animal exposure to Al is uncontrolled due to its widespread use in our lives (Bohrer et al., 2008). Its daily applications include beverage cans, pots and pans, foil, and water filtration. Contaminated food and water are the main sources of Al for humans and animals (Mahmoud & Abdel-Mohsein, 2015). Its residue poses a threat to humans, causing different deleterious effects on the nervous, skeletal, and hematopoietic systems (Domingo, 1995). Al^+3^ can replace Mg^+2^ and Fe^+3^ in the human body and result in cellular growth and communication disturbances as well as neurotoxicity and endocrine disruption (Barabasz et al., 2002; Vardar & Ünal, 2007).

Till now, there has been no Egyptian maximum permissible limit for Al in poultry edibles, but the recommended dietary allowance (RDA) is 60 mg/day for adults (WHO, 1989). In our study, the concentration of Al in poultry muscles and liver was lower than the RDA of the WHO.

The range of Cd concentration (µg/g) was 0.014–0.054 in chest muscles, 0.015–0.088 in thigh muscles, and 0.027–0.104 in the liver of six brands (Table 1). Cd concentration results agree with studies done by Oforka et al. (2012), Nigeria; Cd means in chicken liver and muscles were 0.0457 and 0.0162 µg/g, respectively; Abbas (2017), Cd means (µg/g) in broiler chickens were 0.075, 0.056, and 0.054 in liver, thigh, and breast, respectively; and Reda et al. (2021), Ismailia province, Egypt. Results were lower than the study conducted by Okoye et al. (2011), Enugu State; Cd concentrations were 5.57 and 10.30 µg/g in chicken liver and muscles, respectively; Badis et al. (2014), Cd concentations were 1.39 and 1.49 µg/g in North and South areas of Algeria, respectively; Mahmoud and Abdel-Mohsein (2015), Cd concentations were 1.41, 0.24 µg/g in liver samples and 0.88 and 2.44 µg/g in muscles samples of broiler farms from Assiut and Qena, respectively, and Mottalib et al. (2018), Bangladesh; Cd concentrations mean were 0.243 and 1.092 µg/g in broiler breast and liver, respectively, and higher than the study done by Wang et al. (2020), Jilin Province, China; broiler Cd concentration was 0.003 µg/g.

**Fig. 1:**
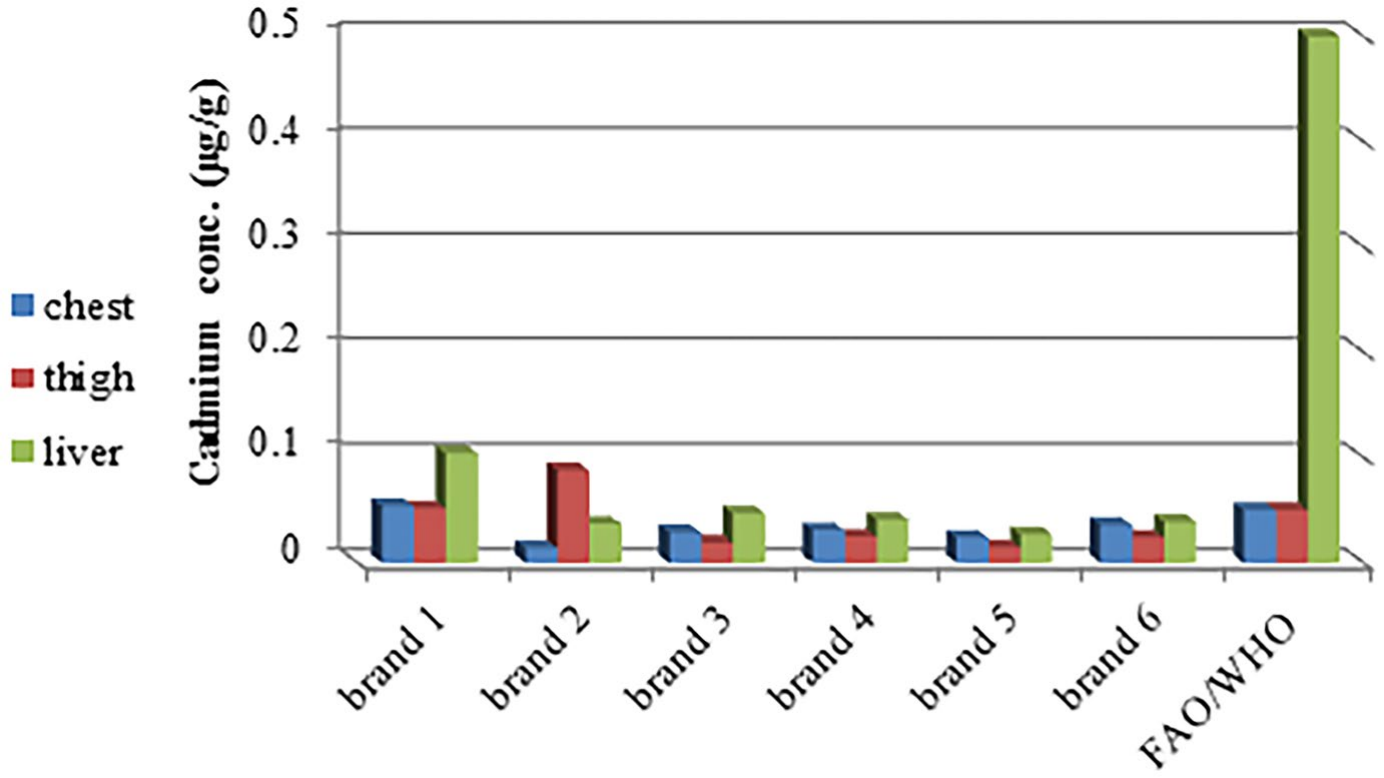
Cadmium concentration (µg/g) in the chest, thigh muscles, and liver in six brands compared with FAO/WHO permissible limits

The presence of Cd in the earth's crust is thought to be its primary route to reach the food chain, and then animals and humans (Rashid et al., 2018). In addition, there are different sources of Cd in our lives, such as fertilizers, sewage, soil, lakes, and groundwater (Chowdhury et al., 2011; Hu et al., 2018). Inhaled or ingested Cd causes respiratory, renal, hepatic, cardiovascular, and skeletal system dysfunction (Mansour, 2014; Rehman et al., 2013), as well as carcinogenesis and mutagenesis (Jaishankar et al., 2014; Jan et al., 2015). Cd levels were below FAO/WHO permissible limits in all samples except the chest muscles of brand 1 and the thigh muscles of brands 1 and 2 (Fig. 1) with 17% (40 muscles and 20 liver); however, all Cd levels during our study were below the Egyptian permissible limits according to EOS.

The range of Pb concentration (µg/g) during the study was 2.560–5.552 in chest muscles, 0.334–1.082 µg/g in thigh muscles, and 0.146–0.952 µg/g in the liver of six brands. Pb residues were significantly higher in chest muscles than thigh muscles and liver (Table 1). Results were in agreement with the study conducted by Mahmoud and Abdel-Mohsein (2015); the Pb mean was 2.75 µg/g in broiler farms and lower than the study done by Okoye et al. (2011), Nigeria; Pb concentrations were 26.29 and 45.05 µg/g in the liver and muscles of different chicken types, respectively; and Badis et al. (2014), Algeria; Pb concentrations were 8.80 and 8.18 µg/g in the North and South areas, respectively. Results in the current study showed that poultry muscle Pb concentration was higher than the liver level, which agrees with the study done by Mahmoud and Abdel-Mohsein (2015) but disagrees with studies conducted by Oforka et al. (2012) and Reda et al. (2021), who showed that internal organs of poultry accumulated more Pb than muscles.

**Fig. 2:**
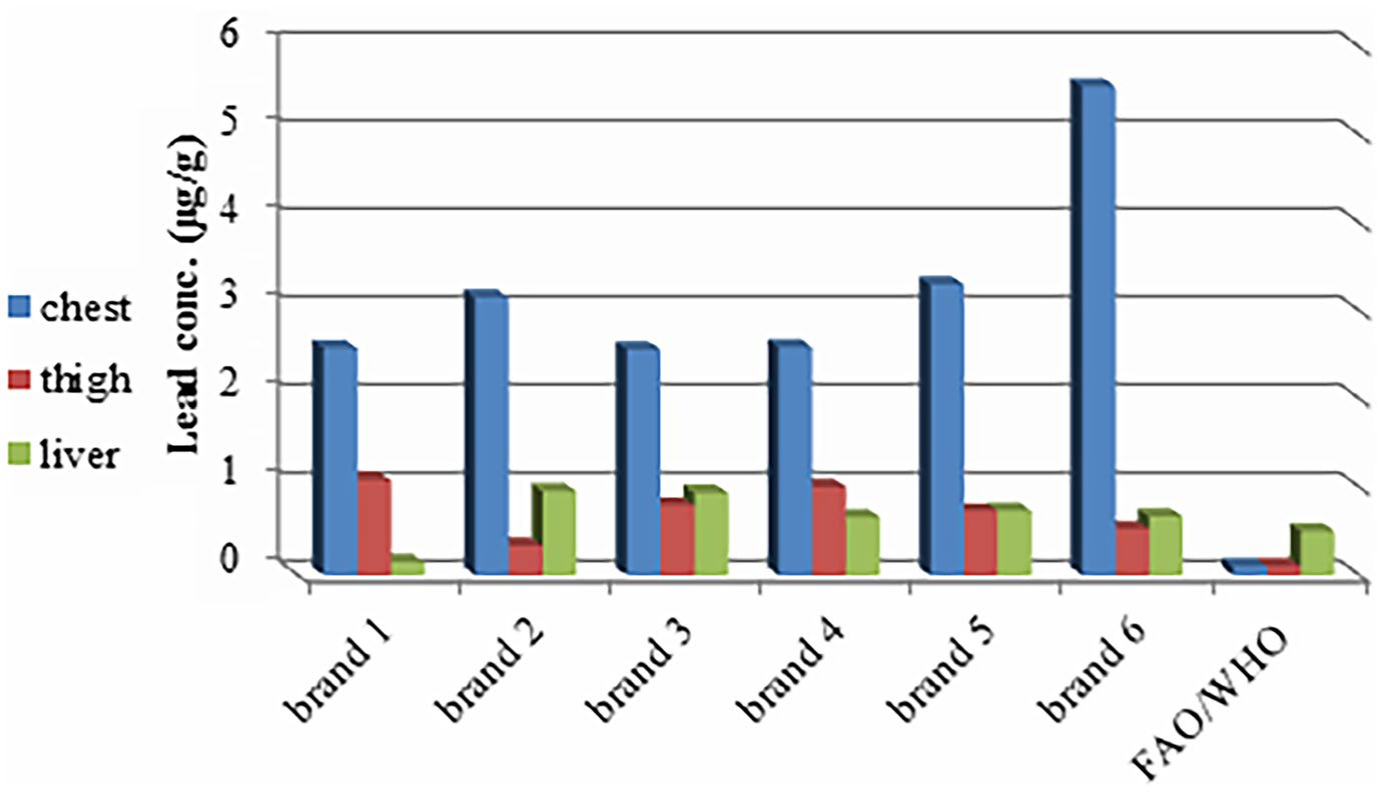
Lead concentration (µg/g) in the chest, thigh muscles, and liver in six brands compared with FAO/WHO permissible limits

Pb is considered one of the most toxic heavy metals and has no benefits for animals or humans. The wide spread of industrial products such as batteries, limestone, leaded gasoline in vehicles, and perfumes, in addition to agricultural and sewage discharges, causes environmental pollution with Pb (EC, 2001; Hussain et al., 2012; Ismail & Abolghait, 2013; Ogwok et al., 2014). Pb can accumulate in the liver and bones as well as affect the nervous, hematopoietic, reproductive, cardiovascular, and adrenal systems (Canfield et al., 2003; Codling et al., 2008; EC, 2001; Khan et al., 2016). In comparison of our Pb results with the previously mentioned FAO/WHO permissible limits, 94% of samples (240 muscles and 100 livers) exceeded permissible limits. All analyzed samples exceeded permissible limits except the liver of brand 1 (Fig. 2). According to EOS, only chest muscles from six brands exceeded permissible limits and represented 33%.

The range of Ba concentrations (µg/g) was 1.971–6.226 in chest muscles, 3.216–6.779 in thigh muscles, and 1.038–4.416 in the liver of different brands (Table 1). It is found in all foods in concentrations ranging from 0.21 to 11 mg/kg (Howe et al., 2005; WHO, 2004; Ysart et al., 1999). Our results were higher than those of studies conducted by Ysart et al. (1999), UK; Rose et al. (2010), UK; Ba concentration was 0.03 µg/g; and González-Weller et al. (2013), whose Ba mean was 0.113 and 0.169 µg/g in chicken breast and liver, respectively.

In addition, Ba is abundant in the earth's crust; its compounds are used in wide applications in our lives, such as fluorescent lamps, paints, bricks, tiles, glass, rubber, oil, and gas industries, as well as barium nitrate and chlorate, which give fireworks a green color. The widespread use of Ba in industrial human activities raises its natural level in the environment.

The range of Bi concentrations (µg/g) was 0.587–3.526 in chest muscles, 0.0043–0.0133 in thigh muscles, and 0.0010–0.0110 in the liver of six brands. Results also showed that Bi residues were significantly higher in chest muscles than thigh muscles and liver (Table 1). Our results disagree with the study done by González-Weller et al. (2013); liver Bi level (53.28 µg/g) was higher than muscle (0.318 µg/g).

Bi is used in the manufacture of shots, shotguns, fishing sinkers, fibers, rubbers, and pharmaceuticals. It is considered one of the less toxic heavy metals and poses a minimal threat to the environment; its toxicity may occur due to high doses applied to burns. There is no evidence that Bi or Ba are carcinogenic, and no permissible limit has been established for either element yet.

The range of Co concentrations (µg/g) was 0.039–0.083 in the chest, 0.014–0.039 in the thigh muscles, and 0.028–0.053 in the liver of six brands. Results also showed that Co residues were significantly higher in chest muscles than thigh muscles and liver (Table 1). Our results agree with the study conducted by Hozan and Hemin (2013): the Co range was 0.00–0.04 µg/g in chicken luncheon that was sold in Sulaymaniah markets; Mottalib et al. (2018), the Co mean was 0.061 and 0.07 µg/g in broiler breast and liver, respectively; and Ersoy et al. (2015), the Co level was determined in the thigh, chest, and liver of poultry around the cement factory in a residential area. On the other hand, the Co range was lower than in the study done by Abdel-Salam et al. (2013), where the Co concentration was 0.2 µg/g.

Co is released into the environment as a result of both natural and anthropogenic activities. It plays a vital role as a constituent of vitamin B_12_, but excessive intake can cause adverse health effects on the endocrine, nervous, and cardiovascular systems (Zahrana & Hendy, 2015). In the previous literature, we have not found Egyptian or international permissible limits for Co in poultry edibles.

The range of Ni concentrations (µg/g) was 0.154–0.228 in chest muscles, 0.143–0.255 in thigh muscles, and 0.112–0.217 in the liver of six brands (Table 1). Our results agree with the study conducted by Mottalib et al. (2018), whose Ni mean in broiler breast and liver was 0.298 and 0.398 µg/g, respectively, but are lower than the study done by Oforka et al. (2012), whose Ni mean was 0.062 and 0.108 µg/g in muscles and liver, respectively, and Mahmoud and Abdel-Mohsein (2015), whose Ni concentration was 4.1 µg/g.

Ni is naturally present in the earth's crust, and the main sources of it in our environment are emissions from refineries, mining, and the combustion of coal, oil, and municipal wastes (USEPA, 2000). Ni is not an accumulated element in the food chain, but it can cause respiratory dysfunction, oxidative stress, allergic skin reactions, hepatotoxicity, immunotoxicity, and cancer (ATSDR, 2004; Das & Büchner, 2007; Lee, 2006). According to the previously mentioned Food and Nutrition Board, WHO/FAO, and EOS safe guidelines for Ni, its concentrations in all samples of different brands examined in our study were far below those limits and considered safe for human consumption.

Fe and Zn were the two highest trace elements in different poultry edible of the study, according to essential trace metal results. The liver accumulated more Fe, Cu, Zn, and Se than the thigh and chest muscles, depending on the brand.

The range of Cr concentrations (µg/g) was 0.266–2.215 in chest muscles, 0.170–0.460 in thigh muscles, and 0.161–1.422 in the liver of six brands (Table 2). Results agree with the study conducted by Iwegbue et al. (2008); the Cr range was 0.01–3.43 µg/g. On the other hand, lower than the study done by Mottalib et al. (2018), the Cr mean was 3.976 and 1.683 µg/ in broiler breast and liver, respectively. Cr is used in dyes, paints, the tanning of leather, the firing of bricks, and metallurgy to impart corrosion resistance and a shiny finish. Due to its wide applications in agriculture, such as fertilizers and wastewater irrigation, people are exposed to it through breathing, eating, drinking, and skin contact (Korish & Attia, 2020). Forms of Cr can be detected by their degree of toxicity; Cr (III) is an essential nutrient for humans and a non-carcinogenic compound, but when they exceed a certain value, negative health effects can occur. Cr (IV) is dangerous for human health and can cause skin rashes, respiratory and heart problems, kidney and liver damage, and cancer (Dębski et al., 2004; Jaishankar et al., 2014; Sahin et al., 2002; Toghyani et al., 2012). The international permissible limit for Cr in edible poultry has been proposed at 1.0 ppm (Youssef A. Attia et al., 2019; National Standard of the People’s Republic of China, 2005; Roychowdhury et al., 2003). According to the previous limit, 11% (20 chest muscle and 20 liver) of the samples in the current study exceeded the permissible limit.

**Table 2:** Essential metals residual concentrations mean (µg/g) in the examined poultry chest, thigh, and liver samples from six brands (*n* = 20 each)

				Brands				
Heavy metal	Organ	g/gµ	1	2	3	4	5	6
Cr	Chest Thigh Liver	Mean ± S.E	0.609 ± 0.024	2.215 ± 0.002	0.283 ± 0.006	0.266 ± 0.005	0.267 ± 0.005	0.296 ± 0.006
			0.460 ± 0.016 a	0.178 ± 0.002a	0.181 ± 0.001a	0.236 ± 0.001a	0.170 ± 0.001a	0.234 ± 0.001a
			0.472 ± 0.013ab	0.161 ± 0.001ab	0.187 ± 0.001ab	1.422 ± 0.088 ab	0.192 ± 0.002ab	0.201 ± 0.001ab
Fe	Chest Thigh Liver	Mean ± S.E	22.835 ± 0.499	8.715 ± 0.238	21.278 ± 0.869	7.631 ± 0.308	8.265 ± 0.385	31.604 ± 0.883
			17.966 ± 0.191a	4.583 ± 0.036a	15.371 ± 0.636a	18.360 ± 0.784a	6.105 ± 0.078a	10.765 ± 0.189a
			38.219 ± 0.201ab	38.585 ± 0.668ab	42.771 ± 0.732ab	48.142 ± 0.804ab	24.151 ± 0.200ab	30.618 ± 0.367ab
Cu	Chest Thigh Liver	Mean ± S.E	0.953 ± 0.011	0.752 ± 0.012	0.989 ± 0.028	0.744 ± 0.020	0.720 ± 0.016	0.736 ± 0.020
			0.806 ± 0.003 a	0.729 ± 0.004a	0.606 ± 0.002a	1.417 ± 0.048a	0.832 ± 0.007a	0.630 ± 0.003a
			4.108 ± 0.010ab	3.221 ± 0.024ab	3.475 ± 0.017ab	3.749 ± 0.025ab	3.084 ± 0.023ab	3.071 ± 0.032ab
Zn	Chest Thigh Liver	Mean ± S.E	11.264 ± 0.193	8.832 ± 0.059	7.963 ± 0.094	13.842 ± 0.478	15.295 ± 0.590	10.205 ± 0.125
			17.272 ± 0.161a	15.635 ± 0.135a	15.171 ± 0.072a	20.131 ± 0.117a	22.792 ± 0.100a	19.358 ± 0.155a
			31.980 ± 0.141ab	22.952 ± 0.084ab	23.643 ± 0.129ab	25.364 ± 0.087ab	24.891 ± 0.119ab	25.992 ± 0.227ab
Se	Chest Thigh Liver	Mean ± S.E	0.335 ± 0.004	0.239 ± 0.001	0.280 ± 0.002	0.284 ± 0.002	0.277 ± 0.001	0.269 ± 0.003
			0.383 ± 0.003a	0.192 ± 0.002a	0.234 ± 0.001a	0.377 ± 0.007a	0.237 ± 0.002a	0.204 ± 0.002a
			0.816 ± 0.003ab	0.627 ± 0.003ab	0.601 ± 0.002ab	0.704 ± 0.003ab	0.659 ± 0.001ab	0.603 ± 0.006ab

The data represent the mean of essential trace metal concentrations ± S.E in poultry chest, thigh muscles, and liver with significantly different values, where (a) indicates a significant difference with chest muscles and (b) indicates a significant difference with thigh muscles at *p* < 0.05

**Fig. 3:**
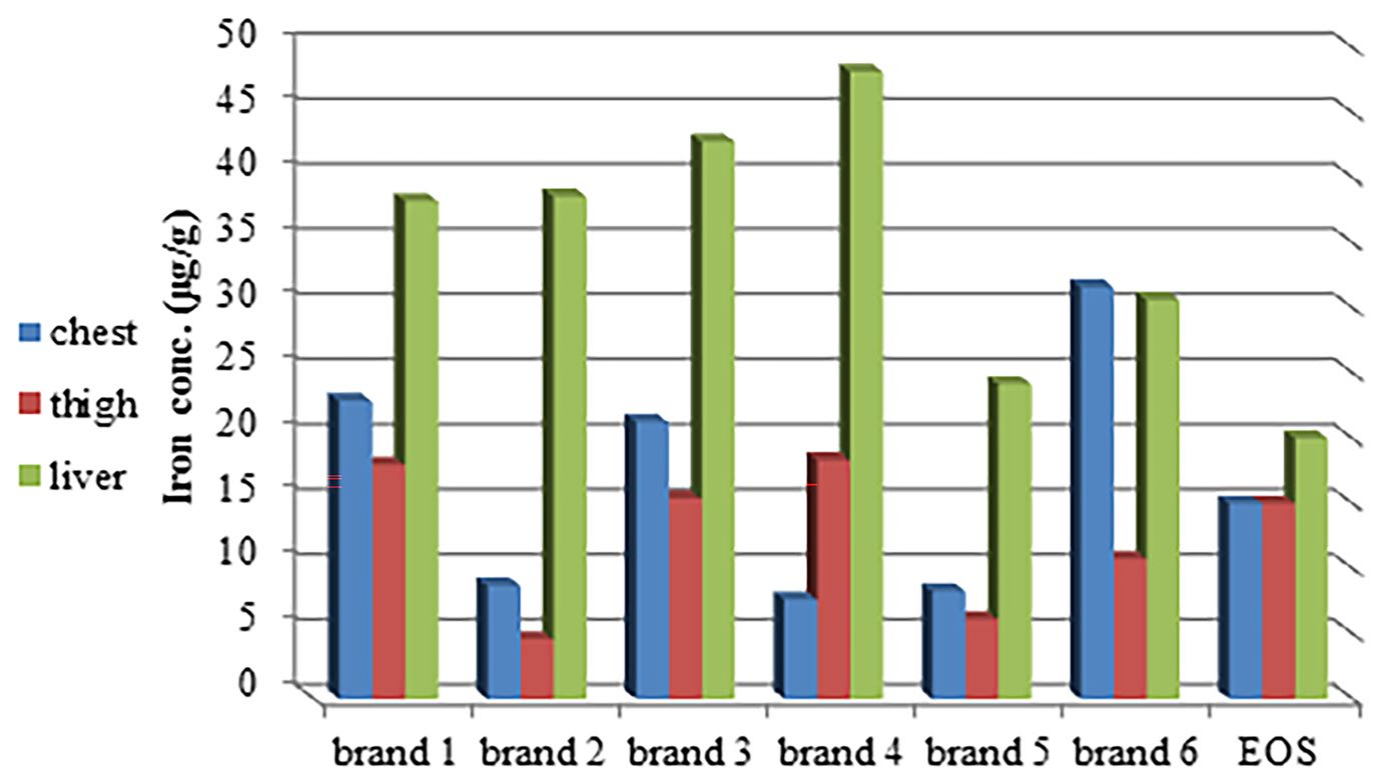
Iron concentration (µg/g) in poultry chest, thigh muscles, and liver in six brands compared with Egyptian Organization for Standardization and Quality Control permissible limits

The range of Fe concentrations (µg/g) was 7.631–31.604 in chest muscles, 4.583–18.360 in thigh muscles, and 24.151–48.142 in the liver of brands (Table 2). Fe results were lower than Badis et al. (2014), Algeria; Fe concentration was 246.83 and 186.33 µg/g in Nourth and South areas, respectively; Korish and Attia (2020); Fe mean was 87.8, 63.1, and 288.2 µg/g in frozen, fresh broiler meat, and liver, respectively, and Reda et al. (2021), Ismailia Province. Thanks to its combination of low cost and high strength, Fe is an indispensable element worldwide. Its applications range from food containers to cars. For humans, it facilitates protein, lipid, and carbohydrate metabolism in the body, but its deficiency causes anemia, a high susceptibility to myocardial infarction, gut infections, and bleeding (Elsharawy, 2015; Jaishankar et al., 2014). High Fe concentrations also cause cardiac arrest and respiratory failure (Korish & Attia, 2020). The Egyptian Organization for Standardization and Quality Control suggested 15.0 μg/g as a safe line for Fe in poultry meat and 20.0 μg/g in offal (EOS, 2010). According to the previous limit, 67% (120 chest and thigh muscles as well as 120 liver) of samples exceeded the mentioned permissible limit (Fig. 3).

The range of Cu concentrations (µg/g) was 0.720–0.989 in chest muscles, 0.606–1.417 in thigh muscles, and 3.071–4.108 in the liver of six brands in the study (Table 2). This study's results were compared with those reported by Okoye et al. (2011), whose Cu mean was 26.29 and 45.05 µg/g in different chicken liver and muscles, respectively; Alturiqi and Albedair (2012)'s Cu range was (2.31–7.79 µg/g). Elsharawy (2015), Cu range was (0.15–1.16 µg/g); Mottalib et al. (2018), Cu mean was 2.422 and 4.092 µg/g in broiler breast and liver, respectively; and Korish and Attia (2020), Cu mean was 0.036, 0.056, and 19.24 µg/g in frozen, fresh broiler meat and liver, respectively. Cu is very common and abundant in our environment due to its high natural presence and its industrial and agricultural applications. It is an essential element for various enzymes in our body, but if levels exceed limits, they cause liver and kidney disorders (Elsharawy, 2015; National Research Council, 1994; Ogwok et al., 2014). Cu does not break down in the environment, so it can accumulate in plants, animals, and soil. Consumption of highly Cu-leveled animal products acts as a threat to public health (Alturiqi & Albedair, 2012; Elsharawy, 2015; Hu et al., 2018; Jaishankar et al., 2014). Its toxic effects extend from the activity of microorganisms and earthworms to human health. The Egyptian Organization for Standardization and Quality Control set 15 μg/g as a safe permissible limit for Cu residues in meat and offal (EOS, 2010). According to the previous limit, all the analyzed samples in the current study were within the permissible limits.

The range of Zn concentrations (µg/g) was 7.963–15.295 in chest muscles, 15.171–22.792 in thigh muscles, and 22.952–31.980 in the liver of brands in the study (Table 2). Zn results agree with studies conducted by Chowdhury et al. (2011), Badis et al. (2014), Algeria, and Hu et al. (2018), China. On the other hand, they were higher than the study done by El Bayomi et al. (2018), whose Zn mean was 2.46 and 4.76 µg/g in broiler muscle and live, respectively. Our results also revealed that Fe, Cu, and Zn concentrations were higher in the liver than in the muscles of poultry, which agrees with results reported by El Bayomi et al. (2018), Hu et al. (2018), and Korish and Attia (2020). The international permissible limit for Zn in edible poultry is set at 20 μg/g (Attia et al., 2019; National Standard of the People’s Republic of China, 2005; Roychowdhury et al., 2003). According to the previous permissible limit, 44% (40 thigh muscles and 120 liver) of our study samples exceeded the mentioned limit.

The range of Se concentrations (µg/g) was 0.239–0.335 in chest muscles, 0.192–0.383 in thigh muscles, and 0.601–0.816 in the liver of brands in the study (Table 2). Our results showed that Se concentrations were higher in the liver than in the muscles of poultry, which agrees with Hu et al. (2018), China. Se is one of the essential trace elements and has a role in metabolism, reproduction, immunological function, and antioxidant balance (Attia et al., 2010; Hosnedlova et al., 2018; Saleh & Ebeid, 2019). The international permissible limit for Se in edible poultry has been proposed at 0.5 μg/g ( Attia et al., 2019; National Standard of the People’s Republic of China, 2005; Roychowdhury et al., 2003), and our results showed that 33% (120 liver) of the samples exceeded the previous limit.

## Health risk assessment

### EDI of metals in poultry muscles and liver

The daily intake of metals was estimated and compared with the RDA and tolerable daily intake (TDI) levels, which are considered good monitoring parameters for human exposure to metals. According to Table 3, the EDI of poultry muscles and liver in the current study was lower than the RDI and TDI (FNB, 2004; FSA, 2009; WHO, 2003), which means that human consumption of poultry edibles with such metal residues had a minimum health risk. The EDI results in the current study agree with those reported by El Bayomi et al. (2018).

**Table 3:** EDI (μg/ kg BW) of analyzed metals in six brands of poultry chest, thigh muscles, and liver

Metal	Poultry part	Estimated daily intake (μg/ kg/day) in different brands						
		1	2	3	4	5	6	IARC
Al	Chest Thigh liver	6.51 7.23 0.0084	4.86 3.23 0.0137	6.98 10.46 0.018	12.41 6.83 0.02	9.23 9.23 0.015	6.18 5.65 0.014	3
Cd	Chest Thigh liver	0.030 0.029 0.000148	0.0079 0.0496 0.0000528	0.0163 0.0107 0.0000671	0.0175 0.014 0.0000585	0.0135 0.00847 0.000038	0.0203 0.0135 0.0000557	1
Pb	Chest Thigh liver	1.455 0.611 0.000208	1.1779 0.188 0.00136	1.49 0.448 0.00133	1.46 0.566 0.000941	1.86 0.411 0.00104	3.13 0.296 0.00095	2B
Ba	Chest Thigh liver	3.51 2.82 0.00148	1.59 1.816 0.00624	1.60 3.828 0.00446	1.423 3.139 0.00015	1.813 1.994 0.00218	1.11 2.344 0.00630	3
Bi	Chest Thigh liver	1.99 0.00355 0.0000157	0.769 0.00242 0.0000147	0.492 0.00389 0.0000152	0.331 0.00751 0.0000131	0.406 0.00299 0.00000142	0.387 0.00474 0.0000145	3
Co	Chest Thigh liver	0.0310 0.0220 0.0000642	0.0293 0.00790 0.0000757	0.0288 0.0220 0.0000571	0.0468 0.0208 0.0000657	0.0220 0.0186 0.00004	0.0378 0.0118 0.0000414	2A
Ni	Chest Thigh liver	0.128 0.144 0.00031	0.086 0.0807 0.00016	0.0999 0.0886 0.000247	0.1016 0.0818 0.000268	0.102 0.106 0.000248	0.120 0.0920 0.000255	1
Cr	Chest Thigh liver	0.343 0.259 0.000674	1.25 0.100 0.00023	0.159 0.102 0.000267	0.150 0.133 0.00203	0.150 0.0960 0.000274	0.167 0.132 0.000287	1
Fe	Chest Thigh liver	12.89 10.14 0.05459	0.4921 2.588 0.0551	12.015 8.680 0.0611	4.309 10.368 0.06877	4.667 3.447 0.03450	17.84 6.079 0.04374	3
Cu	Chest Thigh liver	0.5381 0.4551 0.005868	0.424 0.411 0.00460	0.558 0.342 0.00496	0.420 0.800 0.00535	0.406 0.469 0.00440	0.4156 0.3557 0.00438	3
Zn	Chest Thigh liver	6.360 9.753 0.0456	4.987 8.829 0.0327	4.496 8.567 0.0337	7.816 11.368 0.03623	8.637 12.870 0.0355	5.7629 10.9317 0.03713	3
Se	Chest Thigh liver	0.189 0.216 0.00116	0.1349 0.1084 0.0008957	0.158 0.132 0.000858	0.160 0.2128 0.001005	0.156 0.133 0.000941	0.1519 0.1152 0.000861	3

Estimated daily intake of heavy metals in poultry chest, thigh muscles, and liver in six brands as well as the IARC classification for heavy metals: 1: human carcinogen; 2B: possible human cancer; 2A: probably carcinogenic to humans; and 3: non-carcinogenic to humans.

## THQ and HI of metals in poultry muscles and liver

The THQ and HI were used to assess the non-carcinogenic risks of eating poultry muscles and liver. THQ and HI results were far below 1.0 (Tables 4 and 5), and there was no obvious risk for Egyptian consumers from the current study of poultry muscles and liver, which agrees with the previous studies conducted by Bortey-Sam et al. (2015), Darwish et al. (2018), El Bayomi et al. (2018), and Ogbomida et al. (2018).

**Table 4:** THQ of the analyzed metals in poultry chest, thigh muscles, and liver of six brands

Metal	Poultry part	Estimated THQ in different brands × 10^–3^						
		1	2	3	4	5	6	RfD
Al	Chest Thigh liver	16.275 18.075 0.0210	12.155 8.08812 0.03441	17.4510 26.1646 0.04647	31.025 17.084 0.050017	23.09257 23.09257 0.039053	15.4519 14.1418 0.03626	0.0004
Cd	Chest Thigh liver	0.0304 0.0293 0.000148	0.007906 0.04969 0.0000828	0.0163 0.0107 0.0000671	0.0175 0.014 0.0000585	0.0135 0.00847 0.000038	0.0203 0.0135 0.0000557	0.001
Pb	Chest Thigh liver	0.363817 0.152755 0.0000521	0.44499 0.04715 0.00034	0.361417 0.1120957 0.00033428	0.0365511 0.1416021 0.0002353	0.4660304 0.102778 0.0002617	0.783823 0.0741187 0.0002375	0.004
Ba	Chest Thigh liver	0.050227 0.040393 0.00002118	0.022814 0.029445 0.00008916	0.0229031 0.0546885 0.0000637	0.0203297 0.0448463 0.0000776	0.025904 0.028493 0.0000312	0.015900 0.033487 0.0000901	0.07
Bi	Chest Thigh liver	6.866146 0.012267 0.0000541	2.654157 0.008373 0.0000507	1.699984 0.013436 0.0000527	1.143059 0.025898 0.00004532	1.4001019 0.0103206 0.00000492	1.337788 0.016357 0.00005024	0.00029
Co	Chest Thigh liver	0.1035509 0.0734128 0.0002142	0.0978838 0.0263533 0.0002523	0.0960014 0.0734128 0.00019047	0.156237 0.069648 0.0002190	0.073412 0.0621185 0.0001333	0.1261195 0.03953 0.0001380	0.0003
Ni	Chest Thigh liver	0.0064377 0.0072001 0.0000155	0.0043483 0.0040377 0.000008	0.0049977 0.0044330 0.0000123	0.0050824 0.0040941 0.0000134	0.0051389 0.0053365 0.0000124	0.00604244 0.00460042 0.00001278	0.02
Cr	Chest Thigh liver	0.114637 0.086589 0.0002247	0.4169473 0.0335063 0.0000766	0.053271 0.034071 0.0000890	0.0500713 0.0444241 0.0006771	0.0502595 0.0320004 0.0000914	0.0557184 0.0440477 0.0000957	0.003
Fe	Chest Thigh liver	0.0184217 0.0144937 0.00007799	0.00703069 0.00369726 0.00007874	0.0171657 0.0124003 0.0000872	0.0061561 0.0148116 0.0000982	0.00666766 0.00492511 0.00003928	0.0254960 0.0086844 0.0000624	0.7
Cu	Chest Thigh liver	0.1076345 0.0910319 0.0011737	0.0849330 0.0823353 0.0009202	0.11170048 0.06844337 0.00099285	0.0840294 0.16004002 0.001071142	0.081318857 0.093968457 0.0008811428	0.083125942 0.071154 0.00087742	0.005
Zn	Chest Thigh liver	0.0212031 0.0325124 0.0001522	0.01662518 0.02943102 0.00010929	0.0149893 0.0285576 0.00011258	0.02605591 0.03789421 0.00012078	0.0287910 0.04290322 0.00011852	0.01920969 0.03643913 0.00012377	0.3
Se	Chest Thigh liver	0.037835 0.043257 0.0002331	0.0269933 0.0216850 0.0001791	0.031624 0.026428 0.000171	0.03207577 0.04257945 0.00020114	0.03128517 0.02676745 0.00018828	0.03038162 0.02304034 0.00017228	0.005

The target hazard quotient of the analyzed metals in the poultry chest, thigh muscles, and liver of six brands (1–6), and RfD: the reference dose of the metal (mg/kg/day)

**Table 5:** HI of analyzed metals in poultry chest, thigh, and liver of examined six brands

Poultry part	Estimated HI in in poultry (chest, thigh and liver) of six different brands × 10^–3^					
	1	2	3	4	5	6
Chest Thigh liver	23.99530 18.65821 0.023366	15.939628 8.4238238 0.0365968	19.88135 26.60327 0.048643	32.60215 17.68384 0.052834	25.27498 23.51065 0.040853	17.9558 14.50676 0.038176

Hazard index of metals analyzed in six brands of poultry chest, thigh, and liver

## TR of metals in poultry muscles and liver

TR is a tool used to assess the cancer risk of analyzed metals. The US Environmental Protection Agency accepted for regulatory purposes a cancer risk in the range of 1 × 10^–6^ to 1 × 10^–4^ (USEPA, 2004), and South Africa considered that the individual cancer risk limit was 5 × 10^–6^ (Government of South Africa, 2006). TR results revealed that none of the analyzed metals in the current study pose a carcinogenic risk (Table 6).

**Table 6:** TR of analyzed metals in six brands of poultry chest, thigh muscles, and liver

Metal	Poultry part	Estimated TR in different brands						
		1	2	3	4	5	6	CPSo
Cd	Chest Thigh liver	1.158 × 10^–8^ 1.115 × 10^–8^ 5.6 × 10^–11^	3.0 × 10^–9^ 1.888 × 10^–8^ 1.0 × 10^–11^	6.65 × 10^–9^ 4.07 × 10^–9^ 2.55 × 10^–11^	6.65 × 10^–9^ 5.36 × 10^–9^ 2.2 × 10^–11^	5.15 × 10^–9^ 3.21 × 10^–9^ 1.46 × 10^–11^	7.72 × 10^–9^ 5.15 × 10^–9^ 2.1 × 10^–11^	0.38
Pb	Chest Thigh liver	1.236 × 10^–8^ 5.19 × 10^–9^ 1.77 × 10^–12^	1.512 × 10^–8^ 1.60 × 10^–9^ 1.156 × 10^–11^	1.22 × 10^–8^ 3.8 × 10^–9^ 1.136 × 10^–11^	1.24 × 10^–8^ 4.81 × 10^–9^ 8.002 × 10^–12^	1.584 × 10^–8^ 3.49 × 10^–9^ 8.90 × 10^–12^	2.66 × 10^–8^ 2.52 × 10^–9^ 8.07 × 10^–12^	0.0085
Co	Chest Thigh liver	2.174 × 10^–8^ 1.541 × 10^–8^ 4.5 × 10^–11^	2.055 × 10^–8^ 5.53 × 10^–9^ 5.3 × 10^–11^	2.016 × 10^–8^ 1.541 × 10^–8^ 4.0 × 10^–11^	3.28 × 10^–8^ 1.46 × 10^–8^ 4.6 × 10^–11^	1.542 × 10^–8^ 1.304 × 10^–8^ 2.8 × 10^–11^	2.648 × 10^–8^ 8.30 × 10^–9^ 2.9 × 10^–11^	0.7
Ni	Chest Thigh liver	2.188 × 10^–7^ 2.448 × 10^–7^ 5.27 × 10^–10^	1.478 × 10^–7^ 1.372 × 10^–7^ 2.72 × 10^–10^	1.699 × 10^–7^ 1.507 × 10^–7^ 4.20 × 10^–10^	1.728 × 10^–7^ 1.392 × 10^–7^ 4.56 × 10^–10^	1.747 × 10^–7^ 1.814 × 10^–7^ 4.22 × 10^–10^	2.054 × 10^–7^ 1.565 × 10^–7^ 4.34 × 10^–10^	1.7
Cr	Chest Thigh liver	1.719 × 10^–7^ 1.298 × 10^–7^ 3.371 × 10^–10^	6.254 × 10^–7^ 5.026 × 10^–8^ 1.15 × 10^–10^	7.990 × 10^–8^ 5.111 × 10^–8^ 1.33 × 10^–10^	7.511 × 10^–8^ 6.664 × 10^–8^ 1.015 × 10^–9^	7.539 × 10^–7^ 4.800 × 10^–8^ 1.37 × 10^–10^	1.672 × 10^–7^ 6.607 × 10^–8^ 1.43 × 10^–10^	0.5

Target cancer risk of analyzed metals in six brands of poultry chest, thigh muscles, and liver and CPSo: carcinogenic potency slope (mg/kg body weight/day)

## Conclusion

Although all the analyzed poultry meat and liver were positive for metals (Al, Cd, Pb, Ba, Bi, Co, Ni, Cr, Fe, Cu, Zn, and Se), concentrations were far below the maximum permissible limits except for Pb, Cd, and Fe, which exceeded the EOS and/or FAO/WHO permissible limits. Health risk assessment conducted during the study using EDI, THQ, HI, and TR revealed the safety and minimum health risk for human consumption of metal residues in poultry tissues and liver. Even though THQ and HI values were significantly lower than 1.0 during our study, metal monitoring in poultry products and byproducts is required for human security and safety, especially in Egypt due to a shortage of different sources of red proteins and high poultry meat consumption.

## Data Availability

Is applicable.
